# A multicohort geometric deep learning study of age dependent cortical and subcortical morphologic interactions for fluid intelligence prediction

**DOI:** 10.1038/s41598-022-22313-x

**Published:** 2022-10-22

**Authors:** Yunan Wu, Pierre Besson, Emanuel A. Azcona, S. Kathleen Bandt, Todd B. Parrish, Hans C. Breiter, Aggelos K. Katsaggelos

**Affiliations:** 1 Department of Electrical Computer Engineering, Northwestern University, Evanston, IL USA; 2 Department of Radiology, Northwestern University, Feinberg School of Medicine, Chicago, IL USA; 3 Department of Neurosurgery, Northwestern University, Feinberg School of Medicine, Chicago, IL USA; 4 Departments of Computer Science and Biomedical Engineering, University of Cincinnati, Cincinnat, OH USA; 5 Laboratory of Neuroimaging and Genetics, Department of Psychiatry, Massachusetts General Hospital and Harvard School of Medicine, Boston, MA USA; 6 Department of Computer Science, Northwestern University, Evanston, IL USA

**Keywords:** Machine learning, Intelligence, Magnetic resonance imaging

## Abstract

The relationship of human brain structure to cognitive function is complex, and how this relationship differs between childhood and adulthood is poorly understood. One strong hypothesis suggests the cognitive function of Fluid Intelligence (Gf) is dependent on prefrontal cortex and parietal cortex. In this work, we developed a novel graph convolutional neural networks (gCNNs) for the analysis of localized anatomic shape and prediction of Gf. Morphologic information of the cortical ribbons and subcortical structures was extracted from T1-weighted MRIs within two independent cohorts, the Adolescent Brain Cognitive Development Study (ABCD; age: 9.93 ± 0.62 years) of children and the Human Connectome Project (HCP; age: 28.81 ± 3.70 years). Prediction combining cortical and subcortical surfaces together yielded the highest accuracy of Gf for both ABCD (R = 0.314) and HCP datasets (R = 0.454), outperforming the state-of-the-art prediction of Gf from any other brain measures in the literature. Across both datasets, the morphology of the amygdala, hippocampus, and nucleus accumbens, along with temporal, parietal and cingulate cortex consistently drove the prediction of Gf, suggesting a significant reframing of the relationship between brain morphology and Gf to include systems involved with reward/aversion processing, judgment and decision-making, motivation, and emotion.

## Introduction

Understanding the neural basis of intelligence is a longstanding research domain which has historically aimed at identifying the brain regions involved in various human behaviors, in particular, cognitive tasks. Pioneering work by Binet and Simon found that humans always behaved differently across a broad array of tasks, from naming objects to defining words, drawing pictures, and solving analogies^1^. Spearman synthesized these observations into the hypothesis of a generalized intelligence factor, *g*, thus linking human behaviors to brain functions, which reflects abstract thinking and includes the ability to acquire knowledge, adapt to novelty, develop abstract models, and benefit from schooling and learning experiences^2^. Further work by Cattell^3^ split *g* into fluid intelligence (Gf), which is the capacity to solve novel problems and abstract reasoning, and crystallized intelligence (Gc) which relates to accumulated knowledge^4^. Although Gc and Gf are related and rapidly develop in childhood until adolescence, Gf reaches its steady state during the third decade of life prior to a delayed declination whereas Gc continues developing throughout the lifespan^5^. Of these, Gf has been shown to positively correlate with a vast number of cognitive activities, and to be an important predictor of both educational and professional success^6^. These high-stakes effects of Gf call for an improved understanding of its neural substrate, beginning with an understanding of its neuroanatomical underpinnings. However, how to find the relationship between brain morphology and Gf remains unclear.

Previous work seeking to understand the neural substrates of Gf have focused on a broad array of neuroimaging modalities and lesion models, each of which has its limitations. For example, studies with functional imaging of cognitive tasks, or of synchrony between resting state oscillations in blood-oxygen level dependent (BOLD) signal, have focused on fronto-parietal networks responsible for integrating sensory and executive functions in the form of the parieto-frontal integration theory (P-FIT)^7^. Alternatively, the work combining analysis of brain lesions and imaging have explored how multiple demand (MD) systems might contribute to Gf^8^. Moreover, structural imaging (i.e., morphometry) independent of brain lesions has also evaluated the correlation between brain size and Gf^9^ or evaluated the contribution of specific cortical areas and white matter fiber bundles to Gf^10^, without a theoretical framework. Using these imaging methods, previous studies have identified associations between Gf and cortical morphology such as cortical thickness, cortical area, cortical volume, gyrification and gray matter density^10,11^. However the relative contribution of subcortical structures was not investigated, nor was the relationship between subcortical and cortical brain regions outside of fronto-parietal networks, such as the temporal cortex, which has been implicated in some adaptive processes of insight-based problem solving^12^.

How neural changes are associated with Gf throughout early life is important because it provides valuable information about brain maturation and aging processes, as well as provides insight into the physiological causes of cognitive impairment. Researchers found a strong age-related decline in Gf, which has been recently attributed to white matter differences in the frontal cortex^13^. Furthermore, Kievit et al. suggested that these age-related changes were mediated by both gray matter volume and the anterior forceps^14^. However, due to individual differences in the nervous system and complex age-related brain changes, there is no consensus on this issue. Recently, shape analysis^15^ has shown promise in detecting structural differences across age and behavioral trait groups by analyzing surface geometrical properties. Crucially, these differences are not often detectable through volume changes or gray matter alterations. Thus, surface-based methods may be more sensitive to subtle brain changes related to human behavior and cognition functions^16^. Moreover, neocortical enlargement depends primarily on growth of surface area^17^, which makes cortical and subcortical surface measures important when considering similarities across cohorts with significant age differences. Therefore, this study is going to develop a surface-based method to identify consistent and unique features of brain morphometry related to Gf in different age groups.

Given these considerations, and the dearth of research on both subcortical and cortical contributions to Gf as well as what is common across disparate age groups, the focus of our work was three-fold. *First*, we aimed to identify which brain regions and their morphometric measures were most predictive of Gf. Due to the challenges inherent in modeling all the relevant cortical morphologic features and the limited predictive power of these features, we used a data-driven approach capable of identifying complex non-linear relationships, potentially across remote brain regions, and implicitly encompassing multiple morphometric features such as cortical thickness, cortical area and gyrification, as well as the shape of subcortical structures. The *second* aim of our study was to assess the contribution of the subcortical structures to Gf either alone or combined with cortical morphology. The *third* aim specifically focused on investigating how age, as a surrogate for developmental stage, might be involved in the prediction of Gf. For these purposes, we developed a novel geometric deep learning method capable of extracting relevant cortical and subcortical morphological features. Our method was data-driven and relied on cortical and subcortical surface mesh models, extracted from automated MRI-to-mesh preprocessing pipelines, as an input to graph convolutional neural networks (gCNNs) for inferring Gf. Using a six-fold cross-validation scheme on two large independent datasets of different age groups, we evaluated the robustness of our method and the reproducibility of the predictions across two cohorts with distinct age ranges. Finally, a gradient-based backpropagation method allowed us to map the most predictive cortical and subcortical regions involved in the prediction of Gf.

## Results

This study proposed a new deep learning model using residual gCNNs to predict Gf from cortical and subcortical surface meshes on two large datasets of two different age groups. The performances of three types of gCNNs were evaluated for each dataset, using either: (1) only the inner and outer cortical surface nodes (i.e., Cor), (2) only the subcortical surface nodes (i.e., Sub), or (3) both inner and outer cortical surface and subcortical surface nodes together (i.e., All). Specifically, the inner and outer cortical surfaces refer to the white and pial surfaces derived from FreeSurfer. The mean squared error (MSE) and the correlation coefficient (R) were calculated to assess model performances, which refer to the average squared difference between the predicted Gf and the true Gf, and the strength of a linear relationship between these two values respectively. In addition, to provide interpretability to our model performance, we applied a gradient backpropagation-based visualization method (grad-CAM)^18^ to visualize the brain areas most relevant to Gf prediction and furthermore, calculated spatial correlations between these maps generated by different models.

### ABCD dataset fluid intelligence predictions

**Figure 1:**
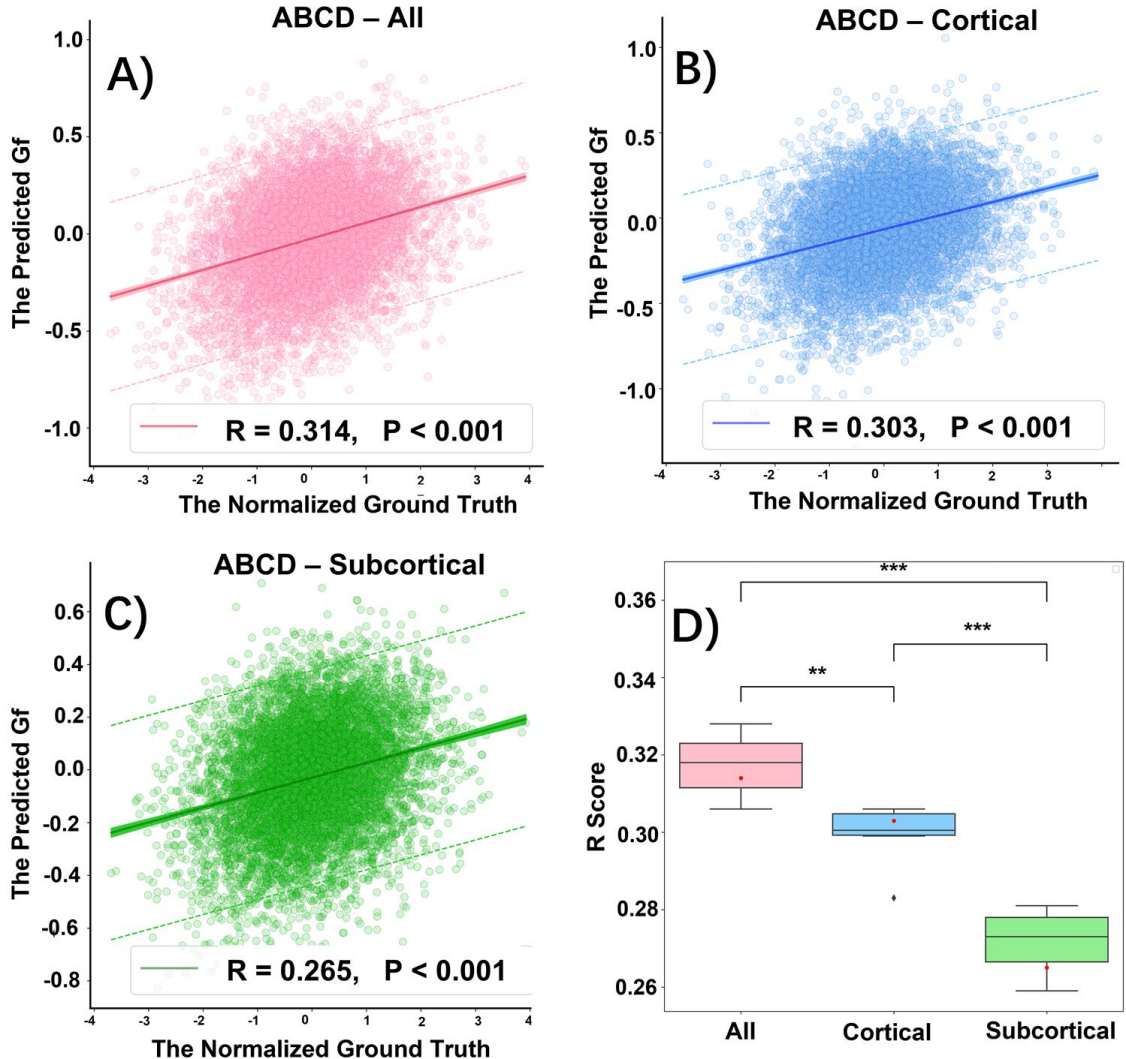
The statistical model performance of predicting fluid intelligence score on ABCD testing dataset. (**A**) All: training with all cortical and subcortical structures. (**B**) Cortical: training with only cortical structures. (**C**) Subcortical: training with only subcortical structures. Significant correlations are found between the predicted Gf score and the ground truth among testing dataset using all structures (**A**), cortical only (**B**) and subcortical only (**C**) respectively. The correlation (R) and *p*-value of the predicted score vs. the ground truth scores are given. The dashed line shows 95% prediction intervals for a new observation and the shaded regions imply the 95% confidence intervals for the prediction population. (**D**) Boxplots compare R scores over all three different datasets across all five folds. The red dots correspond to the mean R score generated from all five folds. (n.s.) Non significant, *p < 0.05, **p < 0.01, ***p < 0.001.

Three models were used based on cortical morphometry, subcortical morphometry, and their combination to predict Gf on the ABCD testing dataset, across six folds. Their comparative performance is shown in Table 1, and Fig. 1A–C show the distribution of predictions for each model. The predictions of all three models were able to significantly correlate with the true fluid intelligence scores. From the result, performance was significantly improved when combining surface data from both cortical and subcortical surfaces (Fig. 1D), which produced an MSE = 0.919 and R = 0.314 [95% confidence interval (CI) 0.308–0.326], followed by using only cortical surface data with an MSE = 0.927 and R = 0.303 (95% CI 0.290–0.309), and only subcortical surface data with an MSE = 0.947 and R = 0.265 (95% CI 0.263–0.281).

**Table 1:** Model performance on ABCD dataset. The models were trained with six-fold nested cross-validation and the predictions were evaluated on the outer testing set of each fold (N = 1345). Time represents the training time of each fold.

ABCD dataset	Testing set								
	All^a^			Cor^b^			Sub^c^		
	R^d^	MSE^e^	Time(s)	R	MSE	Time(s)	R	MSE	Time(s)
Fold 1	0.316	0.913	8143	0.300	0.872	3900	0.265	0.917	1389
Fold 2	0.324	0.911	8290	0.306	0.964	4638	0.281	0.894	1897
Fold 3	0.328	0.946	8452	0.306	0.957	4589	0.279	0.989	1693
Fold 4	0.310	0.913	8340	0.299	0.872	4203	0.275	0.948	1520
Fold 5	0.320	0.908	8502	0.301	0.959	4739	0.259	0.969	1741
Fold 6	0.306	0.957	8601	0.283	0.880	4667	0.271	0.962	1642
Mean ± Sd^f^	0.314 ± 0.008	0.919 ± 0.211	8388 ± 164	0.303 ± 0.008	0.927 ± 0.047	4456 ± 331	0.265 ± 0.008	0.947 ± 0.035	1647 ± 177

^a^All: use both cortical and subcortical nodes. ^b^Cor: cortical nodes only. ^c^Sub: subcortical nodes only. ^d^R: the correlation coefficient measures the linear relationship between the predicted score and the ground truth. ^e^MSE: mean squared error between the predicted score and the ground truth. ^f^Sd: Standard deviation.

### HCP dataset fluid intelligence predictions

**Figure 2:**
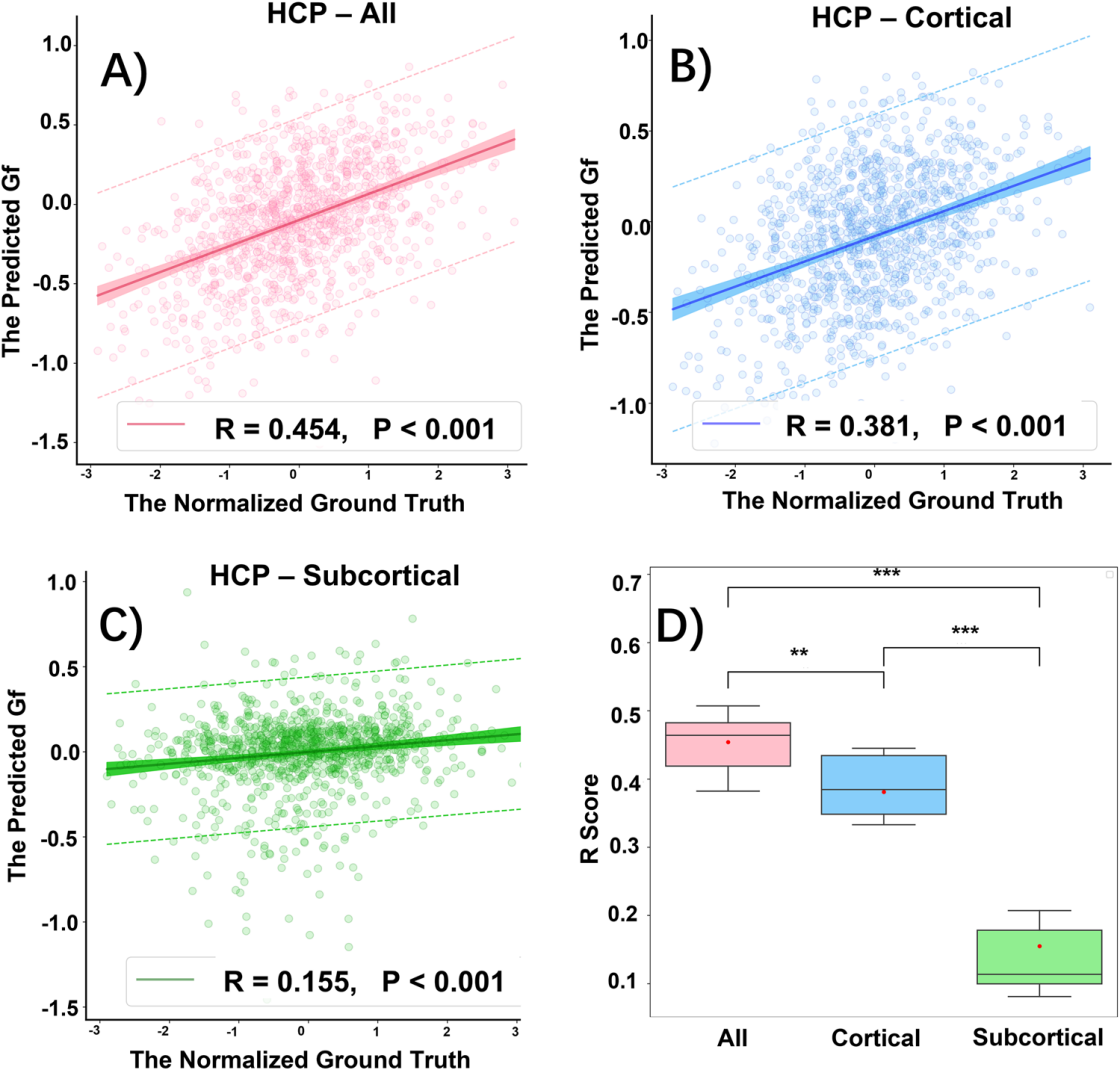
The statistical model performance of predicting fluid intelligence score on HCP testing dataset. (**A**) All: training with all cortical and subcortical structures. (**B**) Cortical: training with only cortical structures. (**C**) Subcortical: training with only subcortical structures. Significant correlations are found between the predicted Gf score and the ground truth among testing dataset using all structures (**A**), cortical only (**B**) and subcortical only (**C**) respectively. The correlation (R) and *p*-value of the predicted score vs. the ground truth scores are given. The dashed line shows 95% prediction intervals for a new observation and the shaded regions imply the 95% confidence intervals for the prediction population. (**D**) Boxplots compare R scores over all three different datasets across all five folds. The red dots correspond to the mean R score generated from all five folds. (n.s.) Non significant, *p < 0.05, **p < 0.01, ***p < 0.001.

The predictive performance for Gf using the HCP testing dataset (Table 2, Fig. 2A–C) has closely approximated findings from the ABCD dataset). Specifically, use of both cortical and subcortical surfaces together in the HCP dataset achieved the best performance with an MSE = 0.834 [95% confidence interval (CI) 0.740–0.929] and R = 0.454 (95% CI 0.400–0.503), significantly outperformed the other two (Fig. 2D). This performance was followed by using only the cortical surface data for an MSE = 0.886 (95% CI 0.784–0.989) and R = 0.381 (95% CI 0.337–0.441) and using only the subcortical surface data alone for an MSE = 1.014 (95% CI 0.906–1.122) and R = 0.155 (95% CI 0.098–0.192). Notably, the overall performance for Gf prediction using both cortical and subcortical structures was better on the HCP dataset compared to the ABCD dataset, whereas the opposite result was obtained if only subcortical structures were used.

**Table 2:** Model performance on HCP dataset. The models were trained with six-fold nested cross-validation and the predictions were evaluated on the outer testing set of each fold (N = 183). Time represents the training time of each fold.

HCP dataset	Testing set								
	All^a^			Cor^b^			Sub^c^		
	R^d^	MSE^e^	Time(s)	R	MSE	Time(s)	R	MSE	Time(s)
Fold 1	0.507	0.809	1969	0.445	0.870	1084	0.197	1.071	540
Fold 2	0.465	0.915	1763	0.405	0.933	997	0.122	1.055	552
Fold 3	0.463	0.889	1856	0.364	0.921	1165	0.105	1.140	531
Fold 4	0.382	0.811	2041	0.343	1.033	1132	0.081	0.852	490
Fold 5	0.404	0.906	1974	0.333	0.774	1057	0.098	0.938	558
Fold 6	0.488	0.677	2845	0.444	0.786	1135	0.207	1.030	533
Mean ± Sd ^f^	0.454 ± 0.049	0.834 ± 0.090	1908 ± 390	0.381 ± 0.050	0.886 ± 0.098	1095 ± 62	0.155 ± 0.054	1.014 ± 0.103	534 ± 24

^a^All: use both cortical and subcortical nodes. ^b^Cor: cortical nodes only. ^c^Sub: subcortical nodes only. ^d^R: the correlation coefficient measures the linear relationship between the predicted score and the ground truth. ^e^MSE: mean squared error between the predicted score and the ground truth. ^f^Sd: Standard deviation.

### Mapping interpretation

**Figure 3:**
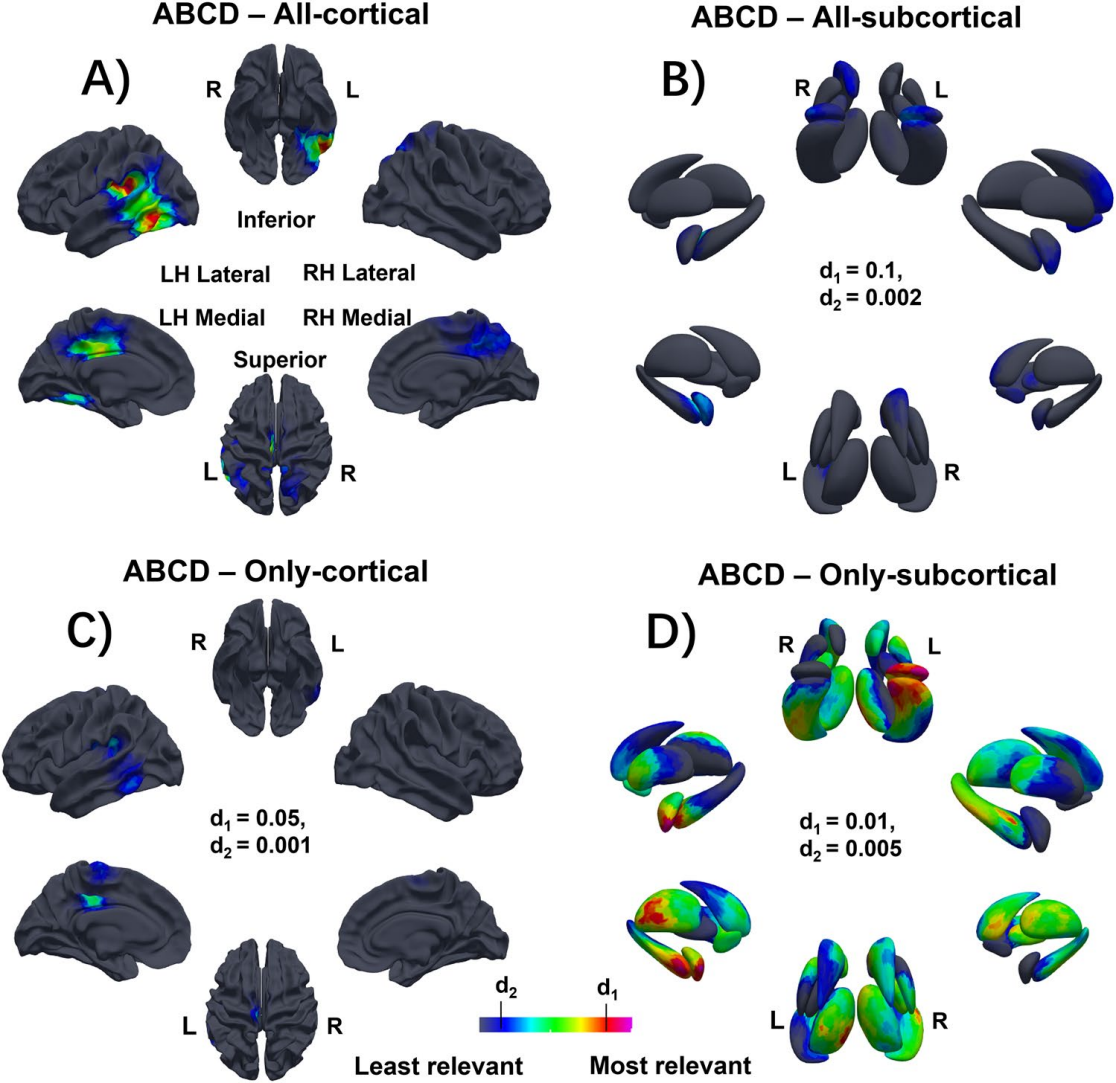
Grad-CAM visualizations to map the brain regions involved in the predictions of fluid intelligence on the ABCD dataset. (**A**,**B**) All: training with all cortical and subcortical structures. (**C**) Only-cortical: training with only cortical structures. (**D**) Only-subcortical: training with only subcortical structures. The red region corresponds to more informative for the Gf prediction. d1, d2: the intensity range of the color map, (**A**) and (**B**) share the same range.

**Figure 4:**
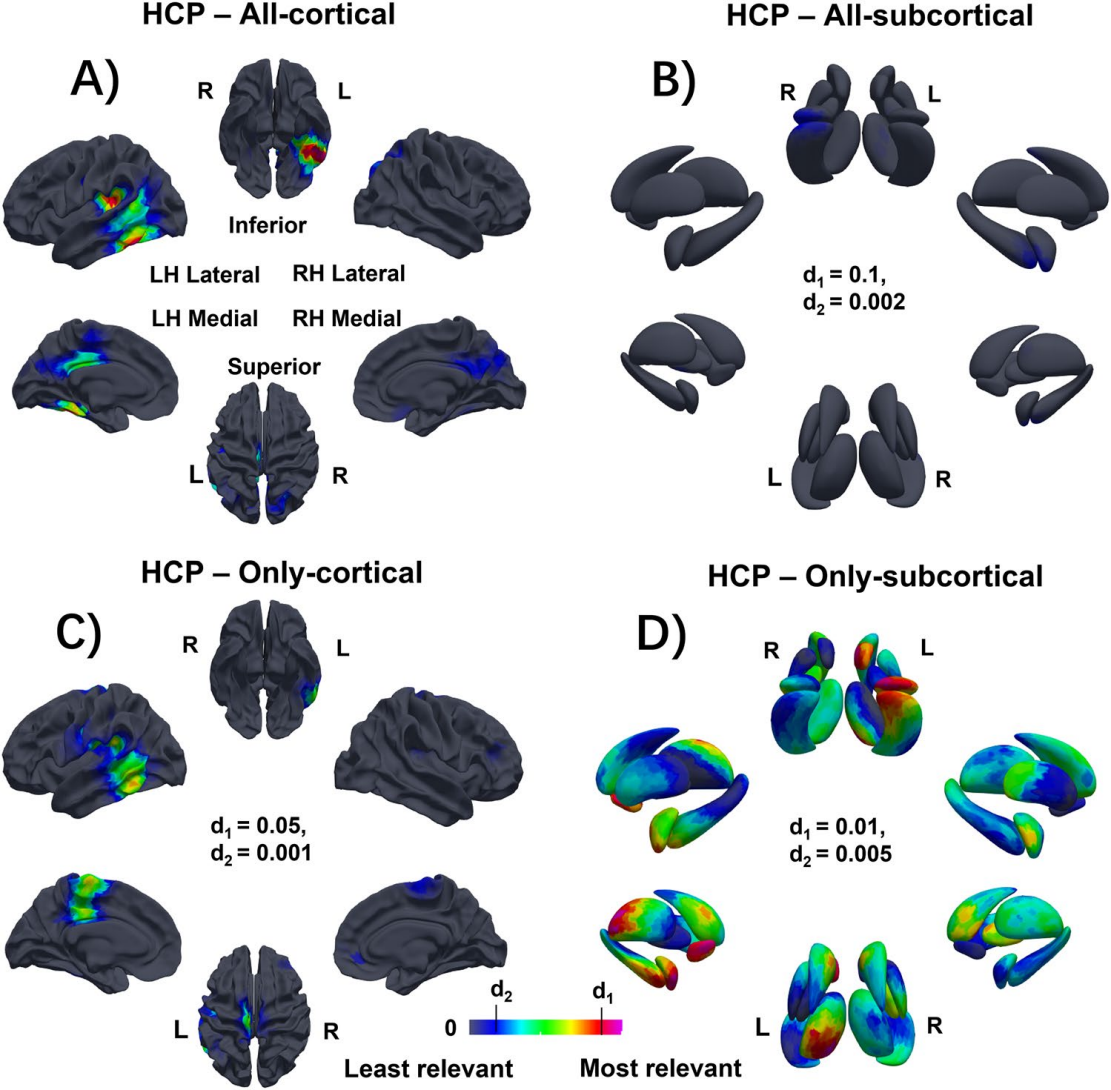
Grad-CAM visualizations to map the brain regions involved in the predictions of fluid intelligence on the HCP dataset. (**A**,**B**) All: training with all cortical and subcortical structures. (**C**) Only-cortical: training with only cortical structures. (**D**) Only-subcortical: training with only subcortical structures. The red region corresponds to more informative for the Gf prediction. d1, d2: the intensity range of the color map, (**A**) and (**B**) share the same range.

Figures 3A–D and 4A–D show the average Grad-CAM maps of the test sets from both the ABCD and HCP datasets that highlight the brain regions involved in the accurate prediction of Gf within each dataset. Figures 3A,C and 4A,C demonstrate that cortical structures play a significant role along with subcortical structures in the prediction of Gf score, which is in keeping with our statistical results. The topographic distribution of relevant brain structures is largely conserved with particular weight placed on the left temporal and parietal lobes in the prediction of Gf across both datasets. Interestingly, the morphology of the left temporal lobe was weighted more heavily in the prediction using the HCP dataset whereas the left parietal lobe was weighted more heavily in the prediction using the ABCD dataset. Other cortical structures including the bilateral paracentral lobules and posterior cingulate gyri were also relevant to the prediction but to a lesser degree. Subcortical structures were more salient in the prediction of Gf from the ABCD dataset and less contributory for the HCP dataset (Tables 1, 2). These subcortical data (Figs. 3B,D, 4B,D) strongly implicate the nucleus accumbens (NAc) and ventral striatum with multiple foci in the pallidum and basal ganglia, along with the amygdala-hippocampus in both datasets. Results from the models using only cortical surface data or only subcortical surface data were similar in distribution but variable in degree when compared to results from the model using both cortical and subcortical surface data together as shown in Supplementary Table S1. Specifically, for the ABCD dataset, the spatial correlation between the cortical maps generated from models using both structures and using only cortical structure was 0.785 (95% CI 0.775–0.794) and the correlation of the subcortical maps was 0.601 (95% CI 0.588–0.614), while for the HCP dataset, the correlation for its cortical maps was higher (0.814, 0.802–0.825) but the correlation of the subcortical maps was lower (0.553, 0.509–0.596).

### Robustness of mappings

Using a six-fold cross-validation, six Grad-CAM maps were generated for each input on the testing datasets. Spatial correlations were calculated within-cohort and across-cohort on the ABCD dataset and HCP dataset to show the robustness of the brain areas involved in the prediction of Gf as shown in Supplementary Table S2. More details of the spatial mapping correlations can be found in Supplementary Fig. S1. Within-cohort mapping correlations ranged from 0.809 to 0.996, where cortical structures in the within-cohort showed higher correlations than subcortical structures on both HCP (Cortex: R = 0.957; 95% CI 0.932–0.965, Subcortical structures: R = 0.809; 95% CI 0.748–0.869) and ABCD datasets (Cortex: R = 0.970; 95% CI 0.961–0.980, Subcortical structures: R = 0.952; 95% CI 0.940–0.964). The across-cohort mapping correlations ranged from 0.721 to 0.814 showing a highly overlapping distribution of important brain areas in prediction of Gf across both datasets.

## Discussion

This study utilized a novel deep learning model using gCNNs with residual connections to infer Gf from cortical and subcortical surface meshes that integrated multiple morphometric features such as cortical thickness, cortical area and gyrification, as well the shape of subcortical structures. It had three aims, to (1) identify the most predictive brain regions involved in predicting Gf, (2) assess the contribution of the subcortical structures to Gf either alone or combined with cortical morphology, and (3) investigate how age, as a surrogate for developmental stage, might be involved in the prediction of Gf. Using two large and independent datasets of pre-adolescent (ABCD project) and young adults (HCP dataset), and a nested six-fold cross-validation scheme, this analysis predicted Gf with significant correlations (R = 0.31–0.45). Across both datasets, as shown in Figs. 3 and 4, the morphology of the left NAc, amygdala and hippocampus, left temporal and parietal cortex as well as the bilateral cingulate cortices consistently drove the prediction of Gf. Given the novelty of these findings, particularly related to the involvement of the NAc, amygdala and temporal cortex, localization was confirmed using grad-CAM to confirm reproducibility across subcortical surfaces and gyral folds.

Divergence between the datasets was observed whereby the left hippocampus and amygdala, left NAc, and multiple foci in the bilateral basal ganglia also played a salient role in the prediction of Gf in the pre-adolescent ABCD cohort. In this dataset, the subcortical structures alone produced an R = 0.27, achieving comparable results with cortical structures alone of an R = 0.3, which indicates the important role of subcortical structures for Gf predictions in ABCD cohorts Together, subcortical and cortical structures produced an R = 0.31. Conversely for the young adult HCP cohort, cortical structures alone produced an R = 0.4, which outperformed subcortical structures alone of an R = 0.1, indicating a major role cortical structures played for adult HCP cohorts when predicted Gf. Likewise, using subcortical and cortical structures together yielded the best performance with R = 0.45. Analysis of the HCP cohort alone identified involvement of the right rectus gyrus in the prediction of Gf which was not seen in the ABCD cohort. In both datasets, significantly better predictions were obtained by combining the cortical and subcortical surfaces suggesting complex, non-linear relationships across remote brain regions at play in Gf prediction. In addition, a substantially larger contribution from subcortical brain structures was identified in the pre-adolescent ABCD cohort compared to the young adult HCP cohort. The finding was in consistent with the results in Supplementary Table S1 that the spatial correlation between subcortical structures was higher on the ABCD dataset (R = 0.60) than on the HCP dataset (0.55), indicating the model trained on subcortical structures alone was more robust for the pre-adolescent ABCD cohort, and therefore more dependent on subcortical structures than the HCP cohort.

### Predictive models of fluid intelligence

Gf refers to the ability to solve novel reasoning problems, which is believed to be independent of experience and education and, as such, believed to be biologically grounded in neurodevelopment^19^. Previous findings have reported an age-related performance in Gf, peaking in late adolescence and declining in adulthood^20^. In this study, we included two datasets of subjects at distinct phases of cognitive maturation. A younger pre-adolescent cohort, the ABCD dataset, included children from 9 to 11 years, an age at which fluid intelligence has not yet reached its putative maximum. In this cohort, we predicted Gf with R = 0.328, which, to our knowledge, improves the prediction accuracy so far reported using this dataset^21–24^. It is challenging to predict Gf on children because their brains are not yet mature. As shown in Table 3, previous ABCD studies reported very weak model performances in Gf predictions (R = 0.01–0.18). Most previous studies manually extracted features on MRIs and applied machine learning methods to make the Gf prediction. For example, using Kernel Ridge Regression classifiers and CNNs, Mihalik et al. used manually extracted voxel-wise brain features (as opposed to automated morphometric analysis) on the ABCD dataset and predicted residualized Gf with an R = 0.031^21^, while Li et al. used XGBoost classifiers on brain volumes and cortical curvatures to predict Gf with an R = 0.18^22^. A recent study developed a fusion deep learning model trained directly on images to predict Gf that combined the slicing features from a 2D CNN with volumetric features from a 3D CNN, achieving an R = 0.1^25^. Our work substantially builds on these ground-breaking reports, while also identifying brain regions, specifically the amygdala and NAc, which has not previously been reported to be involved in Gf.

**Table 3:** Comparisons of the model with the state of the art.

Method	Model	Dataset	Size (n)	Results
Jiang et al.^29^	Task-induced functional connectivity (FC) features + partial least square (PLS) regression	HCP S500	463	R^a^ = 0.409
Greene et al.^27^	Task-induced FC + connectome-based predictive modeling (CPM)	HCP S500	515	R = 0.325
Elliott et al.^28^	General functional connectivity + global signal regression (GSR)	HCP S1200	1043	R = 0.40
He et al.^30^	FC + fully-connected NN	HCP S1200	419	R = 0.31
He et al.^30^	FC features + GNNs	HCP S1200	419	R = 0.15
Pervaiz et al.^31^	Correlation-based FC using Riemannian geometry + data driven parcellation	HCP S1200	1093	R = 0.20
Mihalik et al.^21^	Extracted brain features + kernel ridge regression/CNN	ABCD	8669	R = 0.031
Li et al.^22^	Extracted brain features + XGBoost	ABCD	8669	R = 0.18
Saha et al.^25^	2D/3D Fusion CNN	ABCD	8669	R = 0.1
Our method	Surface information on cortical and subcortical structures + gCNNs	ABCD	8070	R = 0.314
		HCP S1200	1097	R = 0.454

^a^R: the correlation coefficient measures a linear relationship between the predicted score and the ground truth.

A larger number of studies have attempted to predict fluid intelligence using the young adult HCP dataset. It is also challenging because the age of subjects in the HCP dataset ranges from 22 to 35 years old, which corresponds to a different maturational phase when fluid intelligence is close to its full potential^26^. To our knowledge, all previous studies predicting fluid intelligence in the HCP dataset have done so using functional MRI (fMRI)^27–31^. Using functional connectivity analysis of task-based fMRI (FC), Greene et al. reached an R = 0.17^27^. Combining FC with resting-state fMRI (rs-fMRI), Elliott et al. obtained an R = 0.325^28^. Jiang et al. integrated multi-task FC features, applying partial least square regression method to improve the accuracy to an R = 0.409^29^. Our current work is the first to predict Gf on T1 weighted anatomic MRI data alone using HCP dataset, without any behavioral or functional imaging data and it compares favorably with these previously reported state-of-the-art functional imaging methods, by achieving an R = 0.454. Although most of the studies did not share their codes making it hard to have direct comparisons with them, by comparing results using the same metric on the same problem, we were able to show our method was competitive and prominent. Particularly, it suggests there may be advantages from shape mesh representations specific to brain morphometric analysis, boosting performance relative to traditional methods. Our results strongly support an association between brain morphometry and Gf^11^. Moreover, we found that this association was strengthened when both cortical and subcortical structures’ shapes informed our gCNNs, underpinning the interdependencies across remote brain regions that in our review of the literature has not previously been reported.

Overall, our surface-based gCNNs used in this study have several implications for future research. *First*, modeling brain surfaces as input data for gCNNs training reduces the sensitivity of MRI data to different scanner manufacturers, offering good generalizability to other MRI datasets. *Second*, using the coordinates of the surfaces as input features for training massively reduces the input dimension, which saves training time and computations. *Third*, using a surface-based approach to CNN learning offers the potential to map identified relationships between neurocognition and brain anatomy using grad-CAM for visualization. *Finally*, we made our codes and model weights available so that future researchers can easily compare the performance of their models with ours.

### Cortical and subcortical regions involved in the prediction of fluid intelligence

As shown in Figs. 3A–D and 4A–D, the degree of involvement from the temporal, parietal, and cingulate cortices, as revealed by Grad-CAMs, was highly reproducible across folds and displayed remarkable similarities between the two independent datasets, this finding was further supported by the strong spatial correlations calculated across-cohortly between two datasets in Supplementary Table S2. Specific cortical regions for both datasets included the left posterior middle and inferior temporal gyri as well as left basal temporal cortex, left temporo-parietal junction at the posterior aspect of the Sylvian fissure, left posterior cingulate, left interhemispheric paracentral lobule and the right cingulate region. At the cortical level, the only differentiating region between the two datasets was the right rectus gyrus, in which morphometry predicted Gf in the HCP dataset but not in the ABCD dataset. These morphometric findings regarding the temporal, parietal, and cingulate cortices adds complexity to the current framework for understanding Gf, which has mainly focused on involvement of the fronto-parietal networks’ role in combining sensory and executive information^32^ as well as parieto-frontal integration theory (P-FIT)^7,8^. The fact that the temporal, parietal, and cingulate cortices were observed to drive Gf prediction across two independent cohorts raises many questions regarding the strong emphasis placed on the role of the fronto-parietal cortices in Gf by prior studies.

**Figure 5:**
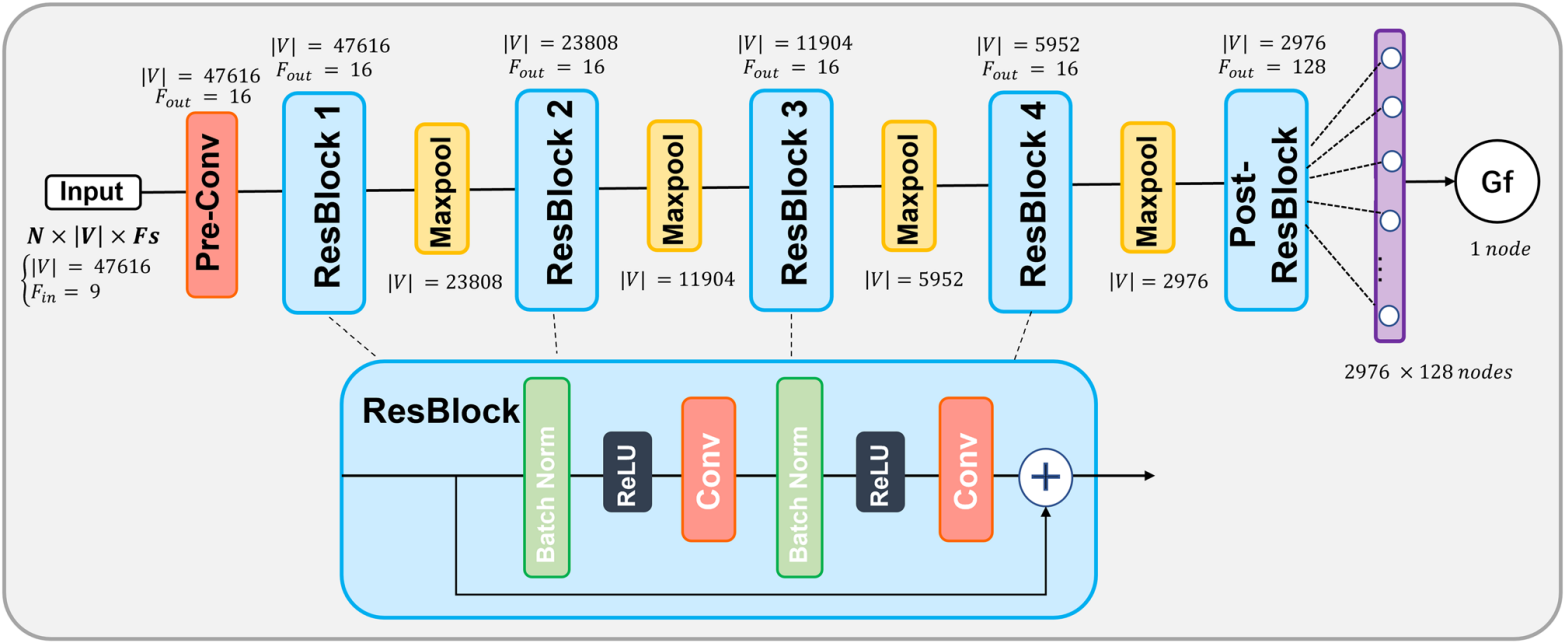
The model architecture. The model contains a pre-convolutional layer, four residual blocks, and a post residual block, followed by a fully connected layer. Each residual block has two subblocks, each with a batch normalization layer, a ReLU activation function, and a convolutional layer. Each residual block is followed by a maxpooling layer to downsample the features. Here, $N$ is the batch size, $|V|$ is the number of vertices, and $F$ is the number of features.

Prior work investigating the neuroanatomic substrate of Gf has identified associations between widespread cortical areas, but relatively few relationships have been reported with subcortical structures. The subcortical structure that has previously been reported to have the strongest association with Gf is the hippocampus. Raz et al. reported smaller hippocampal volume being associated with Gf^33^ while Amat et al. reported smaller hippocampal volume being associated with full-scale intelligence quotient (IQ) and IQ subscales^34^. Others reported hippocampal volume predicting Gf only in musically trained people^35^, and the volumes of hippocampal subfields being more relevant for Gf than working memory^36^, even though working memory has been linked to Gf^6^. Our current findings add to this prior work, particularly in the context of improved Gf prediction resulting from combination of subcortical regions, including the hippocampus, with cortical regions. This work reflects but does not replicate previous reports of an association between Gf and rs-fMRI connectivity between the right hippocampus and medial prefrontal cortex^37^. Our study further indicates how important it is to consider hippocampus morphometry in the context of the morphometry of other subcortical regions, particularly those with minimal association to Gf in the literature, that also have been linked to other aspects of cognitive science, such as reward processing in judgment and decision-making as well as emotion regulation (e.g., NAc and amygdala)^11,38–41^.

A relatively small number of studies have linked Gf to morphometric measures of the basal ganglia, including the caudate and NAc^41^, or suggested that Gf can be segregated from Gc based on NAc volume^42^. Our study adds to these studies by identifying involvement of the bilateral NAc in the prediction of Gf in the pre-adolescent ABCD cohort, and, to a lesser degree, the young adult HCP cohort. The NAc has been a fundamental target of social reward studies and neuroeconomics, with a consensus sentiment that it is a core region for the judgment of value that is fundamental for decision-making^43^. In this context, the NAc has also been considered important for allocation of effort, as with effortful cognitive tasks and motivation^44^, and has been implicated in “grit” or the ability to persevere in a motivated fashion under adversity^39^. The NAc is a critical target of dopaminergic cells in the brainstem^43^, that make it important for motivated behavior, and suggest it would be important for allocating effort to the solution of novel reasoning problems that define Gf.

Related to the function of motivation, and heavily interconnected with the NAc^44^ the amygdala has been considered a core region for emotion regulation, such as the experience and control of fear^40^. To date, we cannot find any studies in the literature that implicate the amygdala with Gf, despite multiple studies implicating other regions with Gf that are contiguous with the amygdala (e.g., hippocampus) or significantly interconnected with it (e.g., NAc). Gf has been implicated with connectivity related to the uncinate fasciculus, a white matter bundle that connects the amygdala and anterior temporal cortex with frontal regions^45^, but not directly connected to amygdala morphometry. Our current findings across two independent cohorts of amygdala morphometry predicting Gf, might be consistent with a role in emotion regulation facilitating the solution of novel problems and adapting learning to new circumstances.

In parallel with considering the location of morphometric changes observed in this study, it is important to consider the complexity involved with morphometry as a field, including the number of independent features measured by voxel-based morphometry, cortical thickness, and volumetrics^10,46,47^. The analysis of the specific contributions of cortical thickness, cortical area and gyrification to Gf can reveal large topological variations depending on the cortical morphometry employed and resulting in sometimes contradictory results that suggest limitations to the specificity of each measurement individually^11,48^. Using a data-driven approach which is agnostic to the individual morphologic features of the brain’s shape, the approach used in this study identified robust and well-localized involvement of both cortical and subcortical regions. The high spatial correlations of the within-cohort and across-cohort mappings in Supplementary Table S2 show the robustness of our models across different datasets. Although the exact nature of the inferred morphometric features is not known using this approach, the network has the ability to identify interactions across individual morphologic features including cortical thickness, cortical area and gyrification, as well as to integrate features related to the shape of subcortical structures in its learning process. It can also take into account subtle and non-linear inter-regional interactions that contribute significantly to an individual’s Gf. Multiple brain regions previously reported in the literature using individual morphologic feature analysis were not revealed to play a role in the prediction of Gf using the current approach. One explanation for this is that our method integrates multi-dimensional interactions across individual morphologic features into its prediction, and the mapped results identified the most relevant brain regions taking these interactions into account.

### Differences in topographic prediction of Gf across age groups

Gf increases rapidly from birth through late adolescence, when it reaches a plateau which is sustained through the third decade of life, followed by a slow decay over the remaining lifespan^26^. This trajectory parallels that of gray matter pruning in the cortex, which is much more pronounced in pre-adolescent children (e.g., ABCD cohort) relative to young adults (HCP cohort). Throughout adolescence, a strong relationship between cortical and subcortical development has been noted with cognitive performance^49^. Stress and emotional strain from adverse familial, educational, and social events over childhood and adolescence can also modulate the rate of growth in Gf^50^. One might thus expect larger inter-subject variability in a younger population when Gf is still in its developmental phase rather than in a young adult population. Our results could be consistent with this interpretation in that we achieved a higher R in predicting Gf for young adults (HCP cohort) relative to pre-adolescent children (ABCD cohort). At the same time, the cortical brain regions involved in the prediction of Gf remained consistent across age groups as revealed by grad-CAM visualization, despite the differences in predictive accuracy. Two other issues also should be noted. Namely, that neurodevelopment impacts the capacity to modulate cognitive behaviors important for Gf^51,52^. Furthermore, subjects from the HCP dataset were all healthy adults while the ABCD dataset included a cross-section of children including those with a broad array of risk factors for developing mental health and addictive disorders, which can impact Gf. Differences in the discrepancy in accuracy across datasets likely represents contributions from a combination of the brain’s developmental trajectory as well as potential cognitive vulnerabilities across the health spectrum^53,54^.

Between these two cohorts, our results showed that subcortical structures played a more prominent role in the prediction of Gf in pre-adolescent children than in young adults. Across both cohorts, only the head of the left hippocampus and the left amygdala consistently contributed to the prediction of Gf. For the younger subjects (ABCD cohort), the right hippocampus and amygdala were also important for the prediction of Gf, along with the left caudate, NAc, and pallidum. The observation of bilateral hippocampi with the ABCD cohort is consistent with suggestions that working memory may be particularly important for Gf in children^55^. In the developing brain, associations between fluid reasoning and subcortical shape have been reported to be widespread, encompassing the bilateral putamen, pallidum and caudate, consistent with our findings^56^. However, our findings involving the left NAc and bilateral striatum are not consistent with other reports of asymmetric right-sided striatal dominance in younger individuals compared to older individuals^57^. Lastly, it warrants noting that medial temporal structures including the amygdala and hippocampus together with the striatum have strong connections to frontal and cingulate cortices^58^, as well as corticostriatal circuits^59^. Through such connections, medial temporal structures and the striatum have been implicated with classically frontal and parietal tasks including executive function^60^ and context coding^61^, which are important processes for adaptation to novelty at play in Gf.

Our study has some limitations. First, while training time is greatly reduced using surface-based gCNNs, it takes now more time to preprocess and convert brain images to graphs using FreeSurfer than before. Some automatic mesh-generation pipelines have been developed^62^, so it would be interesting to apply them to our model in the future to have an end-to-end workflow. Second, we predicted the raw fluid intelligence scores without adjusting for any confounding factors, such as gender, education, and scanner platform. However, although the cross-validation method was used to avoid bias in the dataset, the predictions could still be affected by potential confounding factors. Future work to train models on residualized Gf scores and test the model on additional dataset would better demonstrate the generalization of our model. Finally, although Grad-CAM offers a possibility to inspect relevant brain regions for our model's prediction of Gf, it is challenging to validate this result without adequate human verification^63^. Despite that, we found that the within-cohort and across-cohort Grad-CAM maps were highly correlated, demonstrating the robustness of the model to rely on the same brain areas to predict Gf. Future work could consider retraining the model on data with artificial 'lesions' to validate our findings. Specifically, we expect that if our model depends on the information from specific brain regions, we should observe a deterioration in the new model's performance in their absence. Furthermore, given our work's focus on brain shape, it will be important to consider how to reconcile our findings with previously reported analyses of brain function in future investigations, e.g., as defined by functional connectivity analysis. One alternative would be using spatial gradients of functional connectivity to predict Gf, such as spatial independent component maps of resting-state activity. Another possibility is combining spatial maps of brain function and structure^64^ to predict behavior.

In conclusion, this study shows significant accuracy in the prediction of Gf across two independent datasets using a surface-based gCNN approach on T1 weighted brain MRI data. Across two independent datasets, this study observed that the left NAc, amygdala and hippocampus, left temporal and parietal cortex, and bilateral cingulate morphometry consistently drove the prediction of Gf. Subcortical contributions appeared to be more important for the younger cohort of the two studies, which involved latency stage children (i.e., ABCD cohort) as opposed to late adolescent/young adult subjects (i.e., HCP cohort). The novelty of the amygdala finding and the extensive involvement of subcortical regions that have traditionally been considered reward circuits points to a broader framework for the function of Gf.

## Materials and methods

### HCP and ABCD dataset

Brain MRI and neurocognitive data from two publicly available datasets were used independently in this work: the Human Connectome Project (HCP) S1200 data release and the Adolescent Brain Cognitive Development Study (ABCD) 2.0 release^54,65^. The HCP dataset consists of neurobehavioral measurements and MRI scans from 1097 healthy subjects aged between 22 to 35 years. Subjects were defined as healthy in the absence of diagnosed neurological or psychological conditions. All subjects were scanned on a custom Siemens 3 T Connectome Skyra at Washington University using a standard 32-channel Siemens head coil. The ABCD dataset consists of neurobehavioral measurements and MRI scans from children aged between 9 and 11 years. Subjects from across the United States with diverse health, socioeconomic and ethnic backgrounds were included. Brain MRI data were acquired from three different 3 T scanner platforms: Siemens Prisma, General Electric 750 and Phillips. Further details pertaining to the included subjects, data collection parameters and preprocessing steps can be found on the HCP website^54^ and the ABCD website^65^. Minimally preprocessed T1-weighted MRI scans were obtained from both databases.

In addition to brain MRI data, Gf scores, measured by the NIH Toolbox Neurocognition battery were collected. Specifically, the “*nihtbx_fluidcomp_uncorrected*” variable was included from the ABCD dataset and the “*CogFluidComp_Unadj*” variable was included from the HCP dataset. We chose the raw Gf scores because previous studies have proved that the residualized scores are actually a weakened predictor of intelligence that hinders modeling the covariance between the residual factors and the image-based features^21,66^. This Toolbox Fluid Cognition Composite score was computed by the average of the raw scores from six measures of fluid abilities (the Toolbox Dimensional Change Card Sort Test, the Toolbox Flanker Inhibitory Control and Attention Test, the Toolbox Picture Sequence Memory Test, the Toolbox List Sorting Working Memory Test, and the Toolbox Pattern Comparison Processing Speed Test). The raw Gf scores from two datasets were quantile normalized at first in order to assume the Gaussian distribution of each dataset. Quantile normalization was realized by sorting the scores of each subject from low to high and replacing them with a random standard Gaussian distribution (i.e., mean = 0 and a standard deviation = 1), which was also sorted from low to high. The characteristics of two datasets are summarized in Table 4.

**Table 4:** The characteristics of HCP and ABCD datasets.

	HCP (N = 1097)	ABCD (N = 8070)
Age (mean ± Sd^a^)	28.81 ± 3.70	9.93 ± 0.62
Sex (female/male)	596/501	3861/4209
Fluid intelligence (mean ± Sd)	115.07 ± 11.58	92.25 ± 10.43
Health status	In good health	In different conditions
Scanner	Siemens 3 T Connectome Skyra	Siemens Prisma, General Electric 750 and Phillips

^a^*Sd* Standard deviation.

### MRI data preprocessing

For each subject, inner cortical surfaces (i.e., modeling the interface between gray and white matter) and outer cortical surfaces (i.e., modeling the cerebrospinal fluid/gray matter interface) were extracted using Freesurfer v6.0. Seven subcortical structures per hemisphere were automatically segmented using Freesurfer (i.e., amygdala, nucleus accumbens, caudate, hippocampus, pallidum, putamen, thalamus) and then modeled into surface meshes using SPHARM-PDM. All surfaces were inflated, parameterized and registered to a corresponding surface template using a rigid-body registration to preserve the anatomy of the cortex and subcortical structures^67^. No morphometric evaluation of subcortical structures, re-segmentation, or use of multiple atlases was performed; this study sought to minimize variance from analysis of the feature set used for prediction.

Surface templates were converted to meshes based on their triangulation scheme. Nodes of the meshes were vertices along the surface, and the corresponding edges of the meshes were segments connecting vertices in a triangulation scheme. Overall, the meshes including all structures had 47,616 vertices, 32,768 for the cortical surfaces and 14,848 for the subcortical surfaces. Input features of the network were defined as the Cartesian coordinates of surface vertices in the subjects’ native space resampled into the surface templates. As a consequence, cortical nodes were assigned 6 features (X, Y, Z coordinates of both the inner and outer cortical surface vertices) and subcortical nodes had 3 features (X, Y, Z coordinates of subcortical surface vertices) when they were used for separate training. Therefore, a 9-dimensional vector was assigned to each node of the graph as shown in Supplementary Fig. S4. For nodes of the subcortical structures, the first 3 elements of the input were Cartesian coordinates of the nodes, and the last 6 were zeros while for cortical nodes, the first 3 elements of the vector were zeros, and the last 6 the Cartesian coordinates of the inner and outer cortical surfaces. All coordinate features were normalized into the range of 0–1.

More details about the conversion of the meshes to graphs with hierarchical decomposition and the organization of input features are provided in the Supplemental Material, Figs. S2–S4, and Table S3. All subjects were represented using the same underlying meshes, the features assigned to the vertices were unique to each subject and served as the input to our gCNNs.

To improve the generalizability of our model, we added two data augmentation techniques before inference based on the ad-hoc preliminary analysis: randomized rotations within ± 20 degrees and random Gaussian noise standardized with mean $\mu = 0$ and a standard deviation of $\sigma = 0.02$. The augmentation parameter, ${p}_{a}$, denotes the probability of data augmentation occurring for a single sample. In this study, both datasets used probability of augmentation, ${p}_{a} = 0.5$, indicating that data augmentation was applied with a 50% probability per sample for each iteration of training.

### Spectral convolution on graphs

Convolution operations on meshes can be generalized in the spectral domain, by using the duality property of the Fourier transform for graphs^68^. Specifically, this involves the multiplication of the Fourier transform of a signal on the graph (the vertex features) with the frequency response of the graph, as expressed by the spectrum of the graph’s Laplacian matrix. An undirected graph is defined as $G=\{V,\epsilon ,A\}$, with a set of $|V|=n$ vertices, $V$, and a set of corresponding edges, $\epsilon \subseteq V \times V$, where edge, ${e}_{ij}\in \epsilon$, connects vertex ${v}_{i}$ to vertex ${v}_{j}$. The weighted adjacency matrix, $A \in {R}^{n \times n}$, contains the edge weights for each of the edges in $\epsilon$, specifically, ${A}_{ij}={e}_{ij}$. Since we are considering undirected graphs, $A$ is a square symmetric matrix. The graph Laplacian is defined as $L= D-A$ and its normalized form as $L= {I}_{n}- {D}^{-\frac{1}{2}}A{D}^{-\frac{1}{2}}$, where ${D}_{ii}= \sum_{j}{A}_{ij}$, is the graph’s corresponding diagonal degree matrix, containing the “degree” of each vertex on the graph and ${I}_{n}$ is an $n\times n$ identity matrix. $L$ is diagonalizable via the eigen-decomposition $L=U\Lambda {U}^{T}$, where $\Lambda =diag \left(\left[{\lambda }_{0, } {\lambda }_{1}, \dots ,{\lambda }_{n-1 }\right]\right) \in {R}^{n \times n}$ is the diagonal matrix of eigenvalues and $U=\left[{u}_{0, } {u}_{1}, \dots ,{u}_{n-1 }\right] \in {R}^{n \times n}$ is formed by the corresponding orthogonal eigenvectors ${u}_{i}$.

Let us consider the input feature matrix $X \in {R}^{n \times f}$, where each column vector ${x}_{i} \in {R}^{n}, i=1,\dots ,f$ represents a vector of the $i-th$ feature across all vertices and $f=3, 6, \mathrm{or} 9$ is the number of input features when using subcortical vertices only, cortical vertices only, and both surfaces, respectively. We transform an $x \in {R}^{n}$ to the spectral domain by $\widetilde{x}={U}^{T}x$ and define its inverse transform by $x=U\widetilde{x}$. Therefore, we can define the convolution of any two signals $x$ and $z$, denoted by $*$ in the original space, as the multiplication of their corresponding spectral representations, according to

1 $$y=x \times z = U\left({U}^{T}x \bullet {U}^{T}z \right).$$

A filter can be defined in the spectral domain of the graph Laplacian as a polynomial of order $K$ of the Laplacian, that is, ${g}_{\theta }\left(L\right)= {\sum }_{k=0}^{K-1}{\theta }_{k}{L}^{k}$, with $\theta ={\left({\theta }_{1 }\dots {\theta }_{k}\right)}^{T}\in {R}^{K}$. Then the filtering of a signal $x$ by such a filter is given by

2 $${y = g}_{\theta }(L)x = {g}_{\theta }(U\Lambda {U}^{T})x = U{g}_{\theta }(\Lambda ){U}^{T}x = U{g}_{\theta }(\Lambda ) \widetilde{x,}$$

where $L=U\Lambda {U}^{T}.$ In order to reduce the computational complexity due to the spectral decomposition of $L$, the forward and inverse graph spectral transforms, and matrix multiplications, we approximate the filter ${g}_{\theta }(L)$, using truncated expansions of Chebyshev polynomials of the first kind^60^. That is, the $K$-localized filtering operation is defined as

3 $${g}_{\theta }\left(L\right)= {\sum }_{k=0}^{K-1}{\theta }_{k}{T}_{k}\left(\widetilde{L}\right),$$

where $\widetilde{L}=\frac{2L}{{\lambda }_{max}}- {I}_{n}$, denotes the scaled Laplacian, ${\lambda }_{max}$ the largest eigenvalue of $\Lambda$, $K$ the kernel size (typically $K=3$), and ${\theta }_{k}$ the $k$-th Chebyshev coefficient. The Laplacian is scaled for stability in Chebyshev polynomial operations reliant on $L$, as Chebyshev polynomials for the analogous scalar scenario are defined for stability with inputs in the range [− 1, 1]. ${T}_{k}\left(\widetilde{L}\right)$ is the Chebyshev polynomial of order $k$, which is calculated by ${T}_{k}\left(\widetilde{L}\right)=2\left(\widetilde{L}\right){T}_{k-1}\left(\widetilde{L}\right)- {T}_{k-2}\left(\widetilde{L}\right)$, where ${T}_{0}(\widetilde{L})=I$ and ${T}_{1}(\widetilde{L})=\widetilde{L}$. Finally, the number of trainable parameters per layer is reduced to ${F}_{in} \times {F}_{out} \times K$, where ${F}_{in}$, ${F}_{out}$ are the number of corresponding input and output features. This is analogous to traditional convolutional neural networks (CNNs) where convolutional kernels are used with predefined size (i.e., $K\times K$ kernels for 2D CNNs).

### Network architecture

Figure 5 shows the details of the proposed gCNN architecture. Within our network architecture, we used residual blocks to facilitate the training of deeper networks inspired by Ref.^69^. Using this approach, the output of the previous block is added to the output of the current block to avoid the “vanishing gradient problem” that is likely to occur for deep neural network architectures. Our model contains a pre-convolutional layer (Pre-Conv), four residual blocks (ResBlock), and a post residual block, followed by a single fully connected (Fc) layer with one output that reflects the estimated Gf score. Each residual block has two subblocks, including a batch normalization layer (BN), a non-linear rectified linear unit (ReLU) activation function, and a convolutional layer (Conv). Max pooling layers are used after each residual block to downsample the number of vertices.

**Figure 6:**
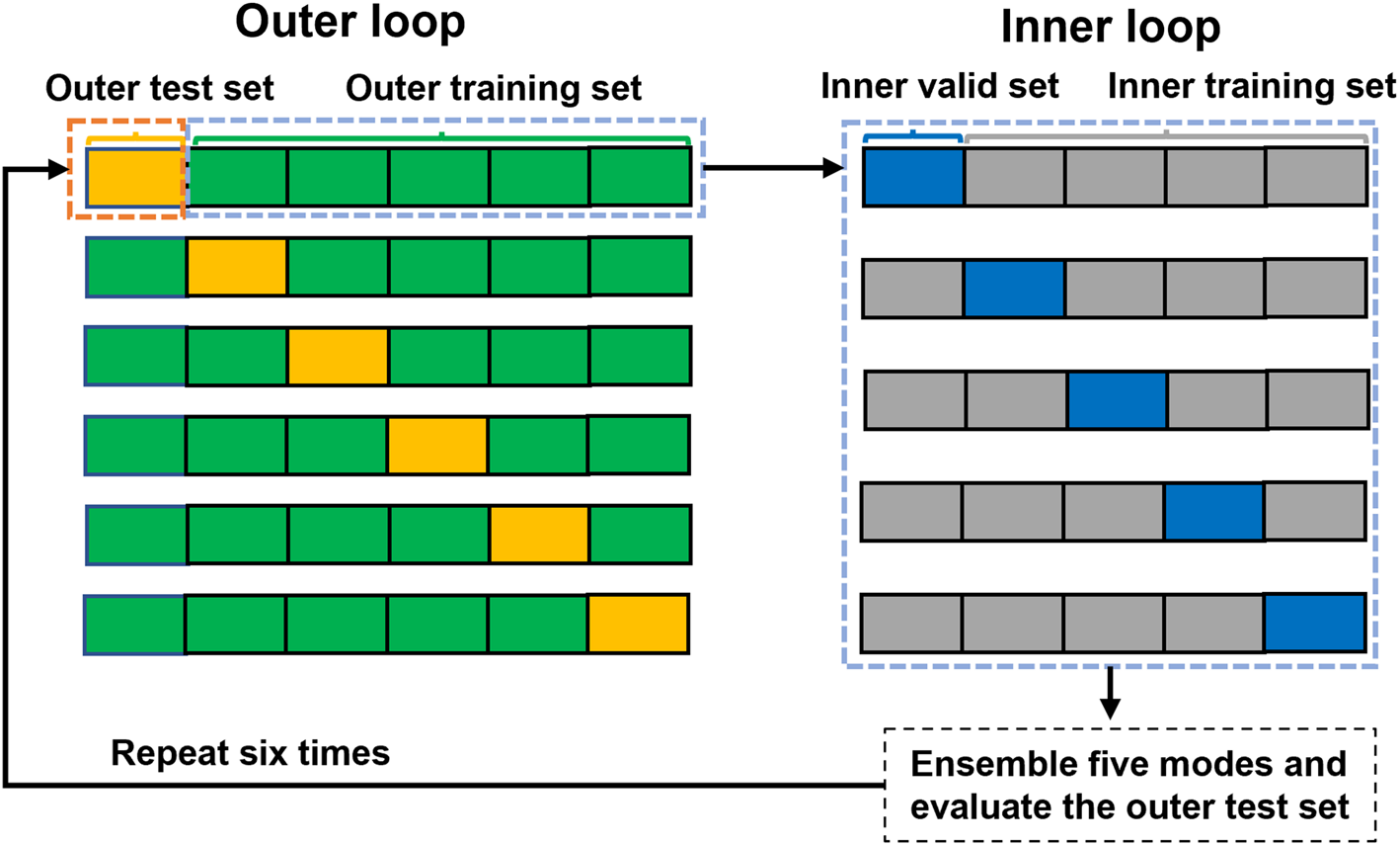
Illustration of the training and evaluation process in six-fold nested cross-validation. The whole process contains an outer loop of six folds and an inner loop of five folds. The model is trained on inner training sets, finetuned on inner validation sets and evaluated on the outer test sets.

### Loss function

The loss function optimized to train our model is composed of three parts: a mean squared error (MSE) term to measure the error between the network’s estimates and ground-truth values, a Pearson’s coefficient of correlation term, $corr$, and an additional regularization term *reg* which is the ${l}_{2}$ norm of the unknown network weights. Therefore, ${L}_{all}$ is defined as

4 $${L}_{all}=MSE+{\lambda }_{1}\bullet reg- {\lambda }_{2}\bullet corr,$$

where $corr$ = $\frac{cov({y}^{p}, {y}^{t})}{{\sigma }_{{y}^{p}}{\bullet \sigma }_{{y}^{t}}}$, with the regularization parameters ${\lambda }_{1}$, ${\lambda }_{2}$ which are adjusted experimentally,${y}^{p}$ is the predicted label, ${y}^{t}$ the ground truth label, $cov$ represents the covariance function of its arguments, and ${\sigma }_{x}$ the standard deviation of $x$. This correlation term is added in order to alleviate the “regression towards or to the mean (RTM)” bias, where the higher the correlation, the lower the loss^70^.

### Grad-CAM visualization

To visualize the most relevant brain areas involved in the network’s decision-making process and to provide some interpretability to our network results, a graphical Gradient-weighted Class Activation Map (Grad-CAM) method was applied to generate a color-coded heat map $$M $$^18^. Grad-CAM uses gradient information flowing back to the last convolutional layer of the model to generate heatmaps highlighting important regions upon which the model focuses and then performs a global average pooling operation to produce the importance weights $${\alpha }^{k} \in {R}^{ k}$$ of each neuron, that is,

5 $${\alpha }^{k}= \frac{1}{N}\sum_{n}\frac{\partial {y}^{p}}{\partial {A}_{n}^{k}},$$

where ${y}^{p}$ refers to the predicted value and ${A}_{n}^{k}$ represents the value at each node $n$ for feature map ${A}^{k}$ at the last convolutional layer. After calculating the weights, $M\in {R}^{n}$ is calculated using a weighted combination of feature maps followed by a $ReLU$ activation function, which is applied to only keep the positive weights and ignore the negative ones, since we are only interested in the features with positive influence on the predicted value of interest. That is,

6 $$M=ReLU\left(\sum_{k}{\alpha }^{k}{A}^{k}\right).$$

Grad-CAM maps were obtained for Gf prediction from each testing set in all six folds. As four pooling layers were used in the model, reducing the number of nodes by a factor 2^4^, we unsampled the generated grad-CAM maps back to the original size using spherical linear interpolation on the cortical and subcortical surfaces in order to overlay the maps back to the original graphs. To compare the distribution of Grad-CAM maps generated by the model using both cortical and subcortical data (All) and the model using cortical only (Cor) or subcortical only data (Sub), we calculated the spatial correlation of these maps across vertices. More details are included in the Supplementary materials.

### Network implementation

A nested cross-validation was used in this work to assess model performance and generalizability, as shown in Fig. 6. The cross-validation contained an outer loop of six folds and an inner loop of five folds. Both datasets were split into six folds, randomly selecting one set as the outer test set and concatenating the rest of the five sets as the outer training set. This whole process repeats six times for each fold. The outer training set, consisting of five folds, was further divided into a validation set (one fold) and an inner training set (the other four folds). This inner process repeated five times and the outer test set was evaluated by an ensembled model averaged from those five trained models. For the HCP dataset, we included 1097 subjects, i.e., in each fold, 914 inner training sets and 183 outer test sets and for the ABCD dataset, we included 8070 subjects, i.e., 6725 inner training sets and 1345 outer test set.

Model performance was evaluated using this nested cross-validation, with the datasets split into six folds each, where each fold was randomly chosen for testing and the remaining five folds were used for training. In each fold, the outer test dataset was evaluated by an averaged model ensembled from all five inner-folds models, and the Grad-CAMs were generated using the average weighted sum on each of the testing subjects. For the ABCD dataset, the networks were trained using a batch size of 32, and a maximum number of 100 epochs. We used the Adam optimizer with a learning rate of $5\times {10}^{-4}$ and a learning rate decay 0.99 every 10 steps. The parameters ${\lambda }_{1}$ and ${\lambda }_{2}$ were both set to $1\times {10}^{-4}$ and the dropout rate of the fully connected layer was set to 0.5. For the HCP dataset, the batch size was set to 50 and the parameter ${\lambda }_{1}$ was set to 0.0005. Due to the smaller dataset size, the maximum number of epochs for the HCP dataset was set to 80. The different network parameters were optimized using our cross-validation and network training was halted when the generalization error increased with the patience factor of 5. The networks were implemented in Python 3.6 using TensorFlow 1.14 and trained using a single Nvidia GeForce 2080Ti GPU.

### Statistical analysis

The mean squared error (MSE), Pearson correlation coefficient score (R), and training time required for each testing fold and for each complete dataset were calculated. The prediction intervals and the confidence intervals were calculated to quantify uncertainty of predictions. A paired t-test was performed to compare the performance of each of the three input types and the p-values were adjusted for multiple comparisons using false discovery rate (FDR), which was considered as statistically significant if the p-values < 0.05. To show the model’s robustness, a spatial correlation (0–1) was calculated on the mapping results (${M}_{c}$) generated from each fold to compare the within-cohort similarity and across-cohort similarity on the HCP and ABCD datasets. More details are included in Supplementary materials. Statistical analyses were performed using scikit-sklearn and NumPy in Python 3.6, figures were generated using Matplotlib and Paraview.

## Supplementary Information

Supplementary Information.

## Data Availability

The raw data set analyzed for the current study is directly downloaded from the Human Connectome Project (HCP) S1200 data (https://www.humanconnectome.org/study/hcp-young-adult/data-releases). Participant recruitment and data collection were provided by Washington University and the University of Minnesota. The raw dataset can be downloaded from their website. We confirmed that all these experiments were performed in accordance with their relevant guidelines and regulations. The pre-processed dataset can be downloaded by the link in https://drive.google.com/drive/folders/1V2Hth1TRtbY3McGU38VfKDtQ_du7-nMQ and more information can be found by the link in https://github.com/YunanWu2168/FluidIntelligence_graphCNN.

## Code availability

The code used in the study is publicly available from the GitHub repository (https://github.com/YunanWu2168/FluidIntelligence_graphCNN).
