# Supplementary material for: A multicohort geometric deep learning study of age dependent cortical and subcortical morphologic interactions for fluid intelligence prediction
Source: Sci Rep. 2022 Oct 22;12:17760. doi: 10.1038/s41598-022-22313-x (PMC9588039; doi:10.1038/s41598-022-22313-x)
Supplement: Supplementary file 1 — Supplementary Information. [file 41598_2022_22313_MOESM1_ESM.docx]

**Supplementary Information for**

A multicohort geometric deep learning study of age dependent cortical and subcortical morphologic interactions for fluid intelligence prediction

Yunan Wu, MS^1*^, Pierre Besson, PhD^2^, Emanuel A. Azcona, MS^1^, S. Kathleen Bandt, MD^3^, Todd B Parrish, PhD^2^, Hans C Breiter, MD^4,5^, Aggelos K. Katsaggelos, PhD^1,2,6^

* Correspondence to Yunan Wu

Contact Email: [yunanwu2020@u.northwestern.edu](mailto:yunanwu2020@u.northwestern.edu)

**This PDF file includes:**

Supplementary text

Figures S1 to S4

Tables S1 to S3

SI References

**Supplementary Information Text**

**Materials and Methods**

**Conversion of meshes to graphs.** Template surface meshes were converted to graphs using their triangulation schemes (Fig. S2). Nodes of the graphs were defined as the surface vertices, and edges of the graphs were triangles segments across vertices. A weight $W_{i,j}$ was assigned to the edge between neighbor nodes $i$ and $j$ such as:

$$W_{i,j}=\frac{e^{\frac{-{d_{i,j}}^{2}}{2}}}{\sqrt{2\pi}}$$

with $d_{i,j}$ is the Euclidean distance between the nodes $i$ and $j$.

**Hierarchical decomposition of the graphs.** High-resolution template graphs underwent a hierarchical dichotomic partitioning using the following steps and illustrated in **Fig. S3**:

1. For each structure, define the root leaf as the graph corresponding to the high-density mesh.
2. Repeat these steps until the average distance across neighbor leaves is less than a threshold $T$
   1. Given a set of leaves at level $L$, partition each leaf at level $L$ into two child leaves at level $L+1$ using spectral clustering ^1^. The new leaves are therefore subgraphs of their parent leaves;
   2. Order the $2^{L+1}$ leaves of level $L+1$ such that the leaves $2\left( i-1 \right)$ and $2i$ are partitions of leaf $i$ at level $L$;
   3. For each leaf of level $L+1$, identify its center node as the node whose betweenness centrality is largest ^2^;
   4. Defines the partition neighbor matrix $M_{L+1}$, of size $2^{L+1}\times2^{L+1}$, such that:

$$M_{L+1}\left( i,j \right)=\left\{ \begin{matrix} W_{L+1}\left( i,j \right) & \text{if leaves }\text{i }\text{ and }\text{j }\text{ have neighbor vertices on the mesh} \\ 0 & \text{if }\text{i}\text{ =}\text{j} \\ 0 & \text{otherwise} \end{matrix} \right.$$

and

$$W_{L+1}\left( i,j \right)=\frac{e^{\frac{-{d_{i,j}}^{2}}{2}}}{\sqrt{2\pi}}$$

where $d_{i,j}$ is the geodesic distance (along the mesh) between the center of the leaf *i* and the center of the leaf *j* at level $L+1$;

- 1. Compute the average distance across leaves’ centers and exit the loop if less than $T$, otherwise continue to step a.

The average distance threshold $T$ was set to 3 mm for the cortical surfaces and 2 mm for the subcortical surfaces. Therefore, the number of decomposition levels for each structure was a function of their surface area. The number of nodes at the finest level is provided in Table S3.

**Aggregation of matrices and features.** Depending on the structures used for the prediction, the matrices $M_{L}$ were block concatenated along the main diagonal to define the whole underlying graph (**Fig. S4**).

When only the subcortical structures were used for prediction, the input features associated to the nodes of the graph were the 3-dimensional Cartesian coordinates of the corresponding center node in individuals’ space $\left[ X_{s}, Y_{s},Z_{s} \right]$. If the cortex only was used to feed the gCNNs, then a 6-dimensional vector was assigned to each node of the graph, containing the Cartesian coordinates of the inner and outer cortical surfaces $\left[ X_{w},Y_{w},Z_{w},X_{p},Y_{p},Z_{p} \right]$. Finally, when both the subcortical structures and the cortex were used to feed the gCNNs, a 9-dimensional vector was assigned to each node of the graph.

**Pooling operation.** By construction of the hierarchical decomposition of the graph and ordering of the node, the pooling operator is applied similarly to a 1-dimensional signal with a stride of 2 and a pooling size of 2. This is conceptually identical to the original gCNNs study ^3^. However, as opposed to the METIS algorithm initially proposed, our approach guarantees that no singleton is ever generated.

**Prediction interval and confidence interval.** The prediction interval predicts in what range a future individual observation will fall, which is defined in Eq. 1, while a confidence interval shows the likely range of values associated with the population mean as defined in Eq. 2.

$CI_{pred} =t_{n-p}^{(\alpha/2)} s_{n}\sqrt{1 + (1/n)}$, (1)

$CI_{mean} =t_{n-p}^{(\alpha/2)} s_{n}\sqrt{(1/n)}$ , (2)

where $t_{n-p}^{(\alpha/2)}$ is a t-statistic with $n-p$ degrees of freedom at the $\alpha/2$ quantile, $s_{n}$is the standard deviation of the observations on sample size $n$.

**Spatial correlation of Grad-CAM maps.** The Grad-CAMs ($M_{c}$) were generated using the average weighted sum on each of the testing subjects. A Pearson correlation (R) was calculated on these maps across spatial vertices to compare the distribution of different maps as defined in Eq.3,

$R = \frac{\sum({M1}_{i}-\bar{M1} )({M2}_{i}-\bar{M2})}{\sqrt{\sum{({M1}_{i}-\bar{M1})}^{2}{({M2}_{i}-\bar{M2})}^{2}}}$, (3)

where ${M1}_{i} and {M2}_{i}$ are values of maps at vertice $i$, $\bar{M1}$and $\bar{M2}$ are mean values of maps. The correlation ranges from 0 to 1, and the closer to 1, the higher the correlation between the two maps. In this study, we calculated the spatial correlation between Grad-CAM maps generated by the model using both cortical and subcortical data and the model using cortical only or subcortical only data. To show the model's robustness, we also calculate spatial within-cohort and across-cohort correlations. Since we used a 6-fold cross-validation in each experiment, six maps were generated for each input, one for each fold. **The within-cohort similarity** refers to the spatial correlation calculated between any two of these six maps to show the robustness within the folds. **The across-cohort similarity** refers to the spatial correlation calculated between the overall maps on the same structure of the HCP and ABCD dataset.

**SI References**

1. von Luxburg, U. A tutorial on spectral clustering. *Stat. Comput.* **17**, 395–416 (2007).

2. Rubinov, M. & Sporns, O. Complex network measures of brain connectivity: Uses and interpretations. *NeuroImage* **52**, 1059–1069 (2010).

3. Defferrard, M., Bresson, X. & Vandergheynst, P. Convolutional Neural Networks on Graphs with Fast Localized Spectral Filtering. in *Advances in Neural Information Processing Systems 29* (eds. Lee, D. D., Sugiyama, M., Luxburg, U. V., Guyon, I. & Garnett, R.) 3844–3852 (Curran Associates, Inc., 2016).


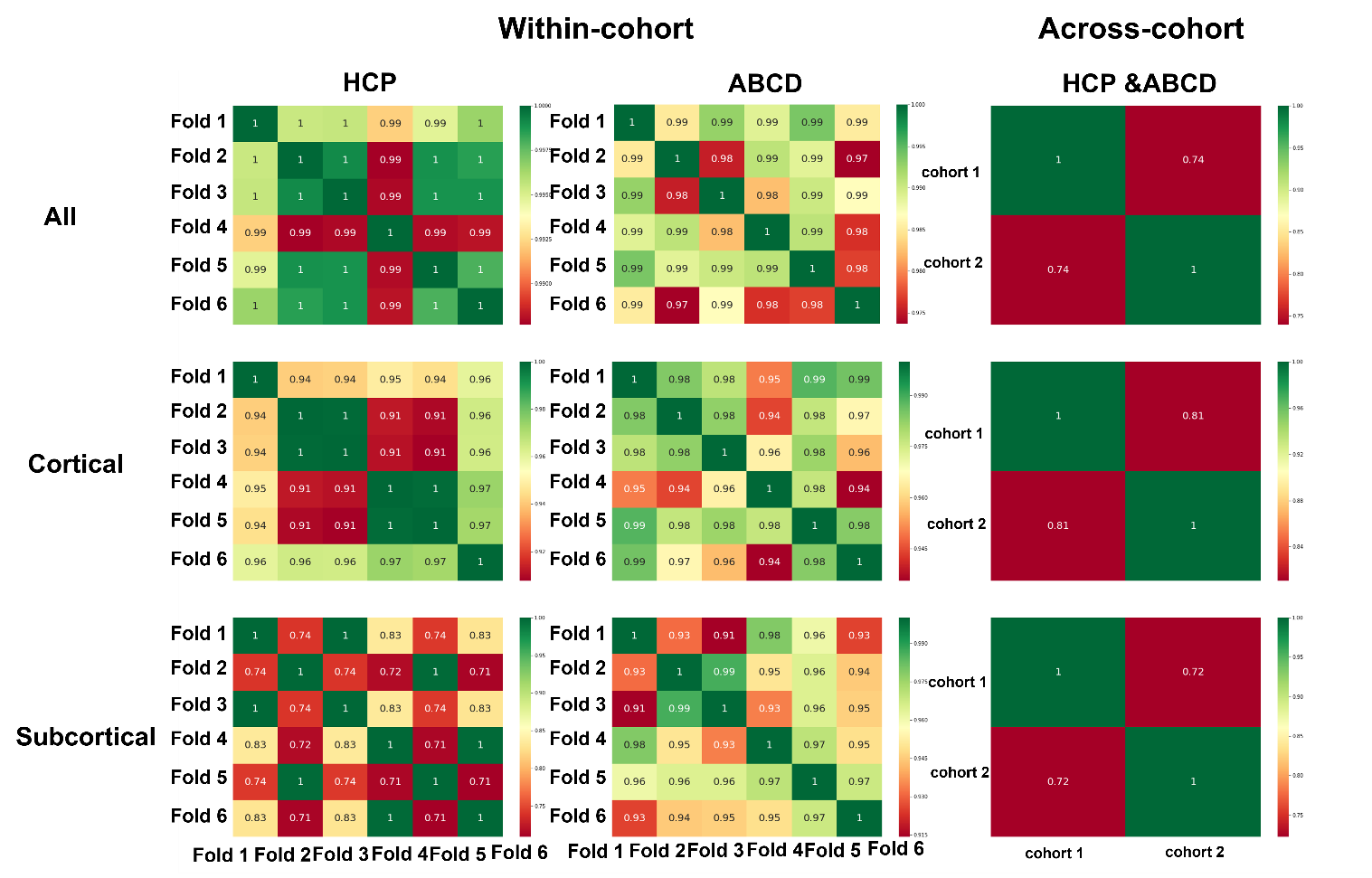


**Fig. S1. The within-cohort and across-cohort correlation details of mappings (Mc) on HCP and ABCD datasets.** The closer to 1, the higher correlations of two group.


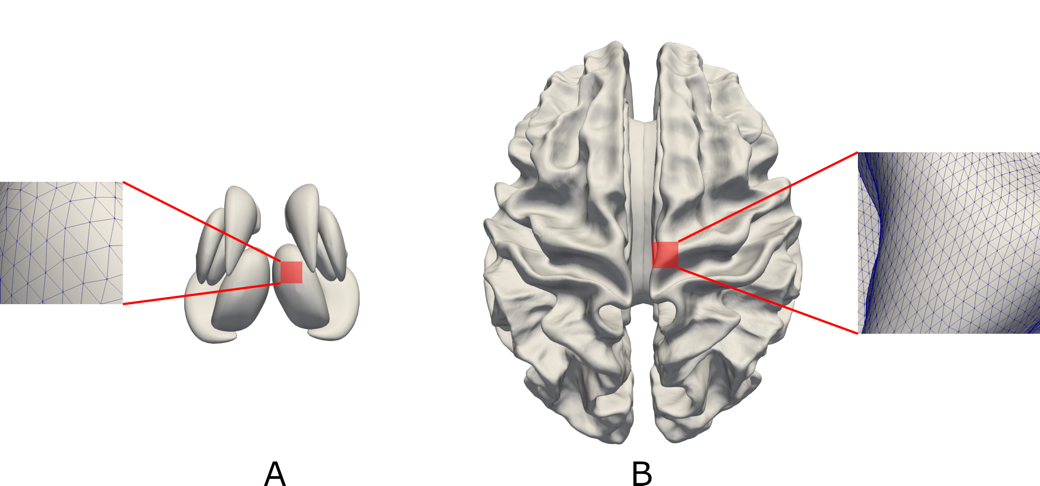


**Fig. S2. Triangulation schemas of surface meshes.** Visualization of the surface template for the subcortical structures (A) and the cortex (B), with close up on their high-density triangulations.


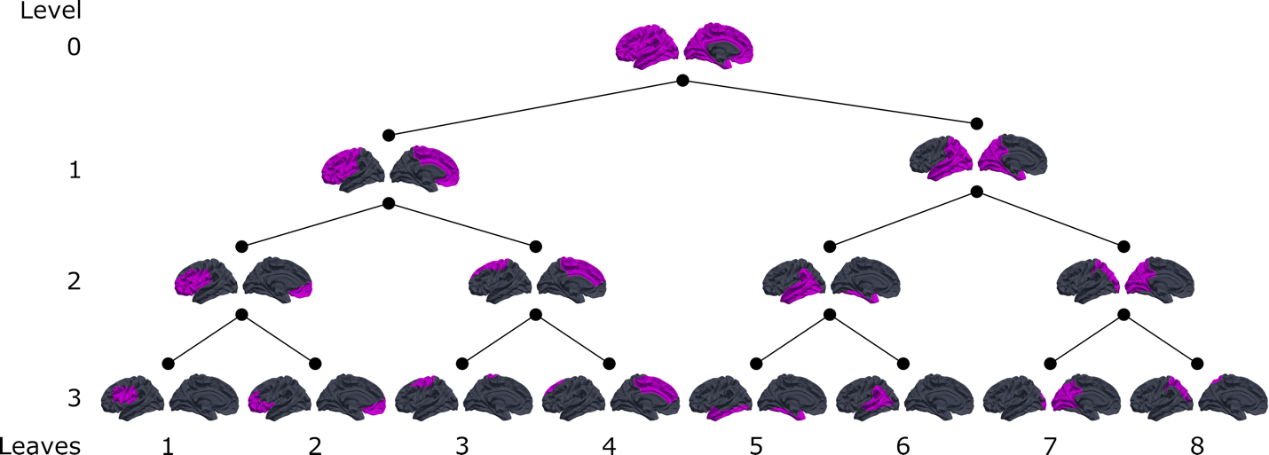


**Fig. S3. First four levels of the hierarchical decomposition of the left cortical surface.** The initial level (0) is the whole structure, in that case the left cortical surface masking out non-cortical regions such as Freesurfer’s medial wall and parahypocampal regions.


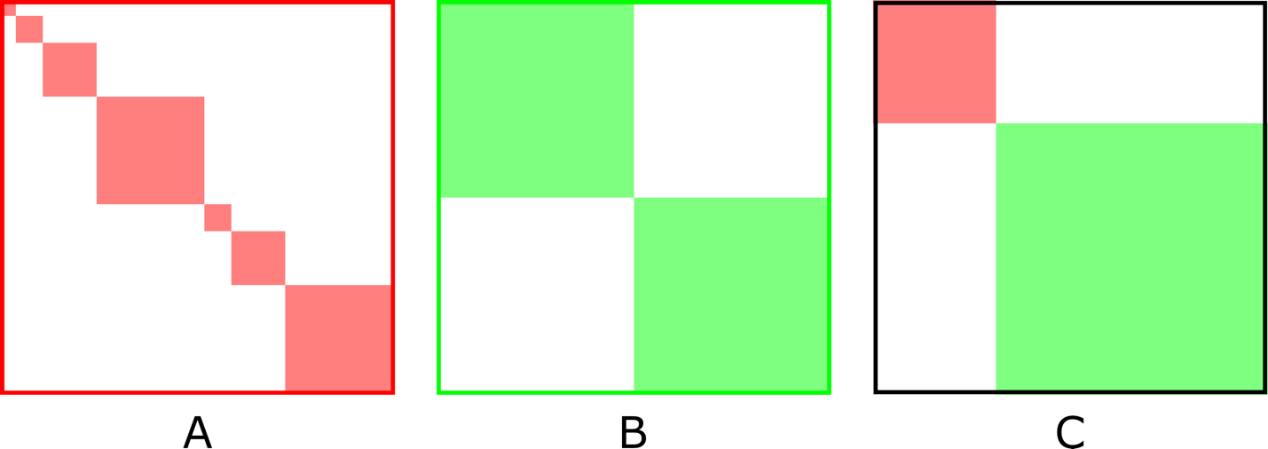


**Fig. S4. Block concatenation of individual structures matrices** $\boldsymbol{M}_{\boldsymbol{L}}$**along the main diagonal.** A) Concatenation of the seven subcortical structures matrices along the main diagonal used for fluid intelligence prediction with the subcortical structures only; B) Concatenation of the left and right cortical hemisphere matrices along the main diagonal for the Gf prediction with the cortex only; C) Concatenation of the subcortical (A) and cortical (B) matrices for the prediction using all the structures.

**Table S1.** The averaged spatial correlations of mappings ($\boldsymbol{M}_{\boldsymbol{c}}$) computed from All-cortical vs. Only-cortical and All-subcortical vs. Only-subcortical on HCP and ABCD datasets across all folds.

| **Spatial Correlation** | **Testing Dataset** | | | |
| --- | --- | --- | --- | --- |
|  | **ABCD** | | **HCP** | |
|  | **All-cortical ^a^**  **vs.**  **Only-cortical ^b^** | **All-subcortical ^c^**  **vs.**  **Only-subcortical ^d^** | **All-cortical**  **vs.**  **Only-cortical** | **All-subcortical**  **vs.**  **Only-subcortical** |
| Fold 1 | 0.793 | 0.601 | 0.823 | 0.511 |
| Fold 2 | 0.771 | 0.620 | 0.819 | 0.523 |
| Fold 3 | 0.796 | 0.596 | 0.802 | 0.515 |
| Fold 4 | 0.783 | 0.612 | 0.827 | 0.609 |
| Fold 5 | 0.780 | 0.587 | 0.810 | 0.586 |
| Fold 6 | 0.785 | 0.590 | 0.801 | 0.571 |
| Mean ± Sd | 0.785 ± 0.008 | 0.601 ± 0.012 | 0.814 ± 0.010 | 0.553 ± 0.038 |

^a.^ All-cortical: The cortical part of mappings trained on both cortical and subcortical nodes. ^b.^Only-cortical: The cortical mappings trained on only cortical nodes. ^c.^ All-subcortical: The subcortical part of mappings trained on both cortical and subcortical nodes. ^d.^ Only-subcortical: The subcortical mappings trained on only subcortical nodes.

**Table S2.** The averaged spatial correlations of mappings ($\boldsymbol{M}_{\boldsymbol{c}}$) on HCP and ABCD datasets across all folds and cohorts.

|  | **Within- cohort** | | **Across- cohort** |
| --- | --- | --- | --- |
|  | HCP | ABCD | HCP & ABCD |
| All ^a^ | 0.996 ± 0.005 | 0.986 ± 0.006 | 0.742 |
| Cor ^b^ | 0.948 ± 0.029 | 0.971 ± 0.016 | 0.814 |
| Sub ^c^ | 0.809 ± 0.106 | 0.952 ± 0.021 | 0.721 |

^a.^ All: use both cortical and subcortical nodes. ^b.^ Cor: cortical nodes only. ^c.^ Sub: subcortical nodes only

**Table S3.** Number of nodes for each structure at their finest decomposition level

| Structure | Number of nodes  Per hemisphere |
| --- | --- |
| Accumbens | 256 |
| Amygdala | 512 |
| Caudate | 1024 |
| Hippocampus | 2048 |
| Pallidum | 512 |
| Putamen | 1024 |
| Thalamus | 2048 |
| Cortex | 16384 |
